# A Heart Rate Variability–Derived Decision Support Tool for Prognostication in Emergency Department Patients With Suspected Infection

**DOI:** 10.1155/bmri/3778740

**Published:** 2026-01-08

**Authors:** Andrew J. E. Seely, Douglas P. Barnaby, Natasha Hudek, Christophe L. Herry, Nathan B. Scales, Shannon M. Fernando, Jamie C. Brehaut, Jeffrey J. Perry

**Affiliations:** ^1^ Department of Thoracic Surgery, Ottawa Hospital, University of Ottawa, Ottawa, Ontario, Canada, uottawa.ca; ^2^ Department of Critical Care, Ottawa Hospital, Ottawa, Ontario, Canada, ottawahospital.on.ca; ^3^ Ottawa Hospital Research Institute, University of Ottawa, Ottawa, Ontario, Canada, uottawa.ca; ^4^ Department of Emergency Medicine, Zucker School of Medicine, Hofstra University, Hempstead, New York, USA, hofstra.edu; ^5^ Department of Critical Care, Lakeridge Health Corporation, Oshawa, Ontario, Canada, lakeridgehealth.on.ca; ^6^ School of Epidemiology & Public Health, University of Ottawa, Ottawa, Ontario, Canada, uottawa.ca; ^7^ Department of Emergency Medicine, Ottawa Hospital, University of Ottawa, Ottawa, Ontario, Canada, uottawa.ca

**Keywords:** clinical decision support, emergency department, heart rate variability, prognostication, sepsis, septic shock

## Abstract

**Aims:**

Prediction of future deterioration in emergency department patients with infection is difficult, and existing prognostic tools are inaccurate. We evaluated the feasibility of deployment of a clinical decision support tool, Sepsis Advisor, which utilizes heart rate variability and laboratory values to predict future deterioration in emergency department patients with treated infection.

**Methods:**

This study was an observational, prospective, Pilot Phase 1 feasibility implementation study involving two sites within a single academic health sciences centre. Then, 71 patients were enrolled, all with suspected/treated infection and systemic inflammatory response. Patients underwent 30 min of electrocardiograph recording. The generated predictive model and Sepsis Advisor report were shown to physicians observationally, > 48 h after clinical encounter, while assessing perceived usability, value, barriers and drivers with using the tool through interviews with nurses and physicians.

**Results:**

Of the 71 patients enrolled, 65 (92%) had adequate duration of heart rate variability measurements to generate a predictive model (average recording: 25 ± 7 min); 100% had clinical data entry. Creatinine, lactate, and INR were drawn 97%, 56%, and 28% of the time and were incorporated into predictive models. Physician and nurse reported drivers for use included potential to facilitate communication, improve care, and ease of integration. Barriers included the need to understand and interpret results from the tool, time constraints, changing routines, and gaining buy‐in. User‐centered feedback informed four improved versions of the tool.

**Conclusions:**

Observational deployment of a heart rate variability–based clinical decision support tool within the emergency department is feasible and perceived to have the potential to improve care.

## 1. Introduction

Sepsis, defined as life‐threatening organ dysfunction caused by a dysregulated host response to infection, remains a major cause of morbidity and mortality globally [1]. Sepsis and septic shock account for 20% of all human mortality, particularly impacting patients in low‐ and middle‐income countries [2]. Delayed recognition of sepsis and lack of timely initiation of antimicrobial therapy are associated with poor patient outcomes, including increasing mortality with each hour of delay [3]. Patients with suspected infection (and possible sepsis) commonly present to the emergency department (ED), with just under a million patient visits per year in the United States [4]. ED clinicians are frequently required to predict the likelihood of future deterioration in these patients in order to make a decision regarding disposition [5]. Some patients with suspected infection may be managed at home, others require admission to hospital wards, and some require admission to the intensive care unit (ICU). Optimizing disposition decision‐making in patients treated for infection is of critical importance, as patients that deteriorate unexpectedly on the ward or at home and then require ICU admission are associated with higher hospital mortality and markedly increased resource utilization [6].

To assist in prognostication of patients with newly diagnosed infection, ED clinicians commonly use the systemic inflammatory response syndrome (SIRS) criteria and the quick Sequential Organ Failure Assessment (qSOFA) [7, 8]. However, both have poor prognostic accuracy [9, 10], are not superior to physician gestalt [11], and qSOFA is not recommended by the Surviving Sepsis Campaign Guidelines [12]. Improved methods for prognostication in this population are needed.

Decreased heart rate variability (HRV) may be used as a physiologic indicator of autonomic cardiac modulation indicating increased stress and/or decreased physiological reserve, which has been shown to accurately predict impending deterioration in patients with infection [13, 14]. Multiple additional studies have demonstrated the prognostic value in HRV in ED patients with infection [15–18], recently undergoing a systematic review [19]. Seeking to implement this predictive information into clinical decision support, we created Sepsis Advisor (SA), a predictive clinical decision support that combines HRV with laboratory tests (lactate, INR, and creatinine) to generate a prediction of the likelihood of future deterioration [14] to assist ED and ICU clinicians with disposition decision‐making.

We conducted a prospective, multicenter mixed‐methods cohort feasibility study to evaluate the feasibility of implementing the SA tool. This observational feasibility pilot was aimed at evaluating patient enrolment, consent, waveform data capture, data entry, report generation by the SA tool, feedback regarding the SA tool, and understanding into provider ease, utility, and barriers to provision.

## 2. Methods

### 2.1. Study Design and Setting

We conducted this prospective cohort study in EDs of two university‐affiliated hospitals within The Ottawa Hospital network (Ottawa, ON, Canada) from August 1/21 to August 1/23. The combined network has 1163 beds and handles over 160,000 emergency visits annually. Each hospital has a combined medical–surgical ICU, with approximately 2500 combined ICU admissions per year. ED physicians make disposition decisions independently and determine the need for ward or ICU admission, requiring admitting physician consultation. All decisions related to ICU or ward admission are made by the admitting service. This study was approved by the Research Ethics Board at The Ottawa Hospital.

### 2.2. Patients

We included adult patients (≥ 18 years of age) presenting to the ED with suspected infection. As in previous prognostic studies [8], “suspected infection” was defined by the following: systemic inflammation (defined by the presence of at least two of the following: temperature > 38.0^°^C or < 36.0°C, heart rate > 90 bpm, respiratory rate > 20 breaths/minute or PaCO_2_ < 32 mmHg, WBC > 12,000 mm^3^, < 4000 mm^3^, or 10% immature [band] forms); and infection is significant enough that the treating physician, triage nurse, or bedside nurse believes that bloodwork is required and has been ordered. Exclusion criteria included any of the following: (1) presence of a do not resuscitate (DNR) or do not intubate (DNI) order prior to study enrollment; (2) inability to perform HRV analysis due to nonsinus or paced rhythm (e.g., permanent pacemaker, atrial fibrillation, supraventricular tachycardia, irregular ventricular bigeminy, atrioventricular dissociation, accelerated junctional rhythm, and other arrhythmias); or (3) met one of the study outcomes within 1 h of ED arrival.

### 2.3. Data Collection

At the time of enrollment, demographic data were recorded, and clinical variables (vital signs and laboratory data) were obtained by clinical research assistants or trained nurses. Enrolled patients were monitored via a Philips MP50 portable bedside monitor on a roll stand using a multimeasurement module, equipped with 5 ECG leads to enable continuous capture of electrocardiography. The Philips monitor was connected via the MIB/RS232 port to a study tablet mounted on the roll stand. Clinical and monitored data were collected using the same software tool loaded on the tablet.

The SA software tool was installed on a study laptop. SA includes (1) a user interface where patient information (demographics, clinical data, and laboratory data) can be entered across several tabs, a recording triggered and a report generated; (2) a capture module that is able to record physiological waveforms (e.g., ECG) from a supported bedside monitor; (3) a processing module that can analyze Lead II of an ECG recording and calculate a preset number of HRV metrics over the duration of the recording; and (4) calculate a set of pretrained predictive models that use the previous HRV metrics and the available laboratory markers (e.g., lactate, creatinine, and INR) to output a probability of deterioration within the next 72 h [14].

For each patient, a SA report was generated digitally, summarizing their risk of deterioration along with relevant clinical information. SA reports were not shown to care providers during care delivery.

### 2.4. Outcomes

Primary outcomes included feasibility of consent, data collection, model calculation, and report generation. The feasibility objectives were determined a priori: 50% of identified eligible patients enrolled and consented; waveform data (> 20 min) captured in > 75% patients; clinical data fully entered in > 90% patients; and SA report (summarizing the clinical information and HRV analysis, requiring a minimum of 15 min of recording) successfully generated with a predictive model score in > 80% patients.

While our stated goal was to capture at least 20 min of waveform data to allow for a more representative HRV analysis, we recognize that this may be hard to achieve in a busy ED and set the minimum required recording duration in SA to 15 min instead, which still allowed us to perform adequate HRV analysis in most scenarios. Moreover, please note that if the recording duration was sufficient (> 15 min) but the HRV analysis was not possible due to excessive noise or poor quality of the recording, the SA tool still generated a report with all relevant clinical information but without the predictive score.

The primary clinical outcome was clinical deterioration within 72 h of ED presentation. We defined “clinical deterioration” as any of the following: ICU admission (for a minimum of 24 h), initiation of noninvasive positive pressure ventilation (for a minimum of 1 h), endotracheal intubation, or initiation of vasopressor or inotropes (for a minimum of 1 h), and/or death. Patients discharged before 72 h of ED presentation were contacted to ensure that they did not attain one of the study endpoints elsewhere

### 2.5. Qualitative Analyses

The perceived usability, value, and barriers and drivers for using the tool in practice were assessed through nurse and physician interviews completed within 48–96 h of the SA report generation. We used a user‐centered design (UCD) approach; an iterative process that is particularly suited to improving the design, presentation, usability (the ease of locating and interpreting information), and usefulness (the right information being presented at the right time) of informational tools and materials [20].

The interview guide included three sections: (1) introductory/icebreaker questions; (2) think aloud of the data entry screens (nurses only) and SA report (both physicians and nurses) based on UCD; and (3) detailed questions about barriers and drivers to implementation, informed by the theoretical domain framework [21] and the consolidated framework for implementation research [22, 23].

Participants included a purposive sample of physicians and nurses whose patients were enrolled in the current study. We focused primarily on ED physicians, since they would be using the tool most frequently, but also included physicians from consulting services (e.g., internal medicine) who might be involved in disposition decision‐making. All participants provided consent to be interviewed.

Interviewees were sent an invitation to participate by email, followed by up to two reminders for nonresponders at 1‐week intervals. Interviews were conducted virtually or by telephone. All interviews were audio‐recorded unless the participant declined. A trained psychologist with over 10 years of interview experience (NH) conducted all interviews. Prior to the interview, none of the nurses or physicians had seen the generated SA report. Participants were offered a $25Can gift card as a token of appreciation for their time.

To facilitate iterative updating of the tool, analysis was based on interview notes, with coders only revisiting recordings when clarification was needed. A single coder compiled all UCD‐related feedback into organized reports for review by the study team. Changes were made iteratively as the SA tool was developed. Two to three interviews are generally sufficient to identify actionable improvements before further interviews are conducted. This process was repeated until no new/major suggestions to improve the tool were noted. Barriers (Bs) and drivers (Ds) to implementation (B/D) coding focused on the anticipation of mediating factors to using the tool in practice. Coding for the B/D portion of the physician and nurse interviews was performed inductively from interview notes. Themes and subthemes were developed initially by a single coder. With each new interview, themes and subthemes were revised, combined, and separated as coding progressed and reviewed periodically by study team members.

## 3. Results

### 3.1. Patient Characteristics and Outcomes

We prospectively included 71 patients with suspected infection in the ED. Patient characteristics are shown in Table 1. Mean (SD) age was 61.3 years (16.0), and 51% were female. The most common source of infection was pulmonary (35%), followed by genitourinary (17%) and gastrointestinal (9%). Source of infection was unknown in 21 (30%) patients. EKG recordings for the HRV assessment were performed 5.4 h after ED presentation on average (SD: 2.3 h). Bloodwork was ordered prior to the HRV assessment and was typically available less than 3 h after the HRV assessment. Patient outcomes and feasibility metrics are shown in Table 2. A total of four patients (6%) met the deterioration outcome (see definition above) within 72 h. Of these, two were admitted to the ICU (4 and 9 days after the HRV assessment, respectively); one died in hospital (5 days after), and one was readmitted to the hospital (3 days after).

**Table 1:** Demographics, suspected source of infection, and comorbidities.

**Demographics**	**Total** **n** = 71
Age	61.3 (16–89)
Sex	36 female (51%)
Patients with two or more SIRS criteria	68 (96%)
Patients admitted	53 (75%)
Patients with ventilatory O_2_ on admission	14 (20%)
COVID‐positive patients	4 (6%)
qSOFA (median (IQR))	17 (42)
SOFA (median (IQR))	11 (13.5)
Length of stay (days)	6.3 (0.2–69.1)
Suspected source of infection	
Pulmonary	25 (35%)
Genitourinary	12 (17%)
Gastrointestinal	6 (8%)
Skin/soft tissue infection/osteomyelitis	2 (3%)
Head and neck	2 (3%)
Viral syndrome	2 (3%)
Hepatobiliary	1 (1%)
Unknown	21 (30%)
Comorbidities	
Smoking history	15 (21%)
Cardiac illness	10 (14%)
Severe cardiac illness	2 (3%)
Respiratory illness	19 (27%)
Severe respiratory illness	1 (1%)
Liver illness	2 (3%)
Hypertension	19 (27%)
Immunocompromised	13 (18%)
Chronic kidney disease	6 (8%)
Acute kidney injury	2 (3%)
Receiving chronic dialysis	1 (1%)
Diabetes, insulin dependent	6 (8%)
Diabetes, oral hypoglycemics	4 (6%)
Diabetes, diet controlled	4 (6%)
Cancers	11 (15%)
Other	≤ 2 (3%)

Abbreviation: SOFA, Sequential Organ Failure Assessment.

**Table 2:** Patient deterioration outcomes.

**Outcomes**	
Hospital readmission	1 (1%)
Intubation	1 (1%)
Intensive care unit admissions	2 (3%)
Noninvasive ventilation	2 (3%)
Vasopressors/inotropes	1 (1%)
Death	1 (1%)
Any outcome	4 (6%)

With regard to feasibility (see Table 3), consent rate was 79% among patients approached. Of the 71 patients included in the study, 65 (92%) had a lead II ECG waveform of adequate duration and quality for HRV analysis. Average duration of recording was 25 min (SD: 7, range: 15–40). Six patients had very poor‐quality recordings (i.e., an extremely noisy ECG Lead II recording where heart beats could not be reliably detected for most of the recording duration). Clinical data was successfully entered in the user interface of SA in 100% of patients. Laboratory values (e.g., creatinine, lactate, and INR) ordered for the patients were up to the ED physician′s discretion (i.e., not prescribed in this observational study), varied across patients (see Table 3). Then, 97% of patients had a creatinine, 56% had a lactate, and 28% had an INR test performed.

**Table 3:** Feasibility outcomes.

**Feasibility metrics**	
Consent rate	79%
Total number of patients enrolled	71
Patients enrolled at Site 1 (general campus)	53
Patients enrolled at Site 2 (civic campus)	18
Patients with useable heart rate recording	65 (92%)
Average recording duration (minutes)	25 (min: 15, max: 40)
Recordings with less than 20 min	24 (34%)
Patients with creatinine	69 (97%)
Patients with lactate	40 (56%)
Patients with INR	20 (28%)
Patients with respiratory rate	71 (100%)

Based on the availability of any or all of the laboratory markers to add to the basal model of HRV, five predictive models were utilized, all using the same methodology and dataset as previously described [14]. See Table 4 for predictive models and specific HRV metrics and labs used.

**Table 4:** Predictive models.

**Model**	**Heart rate variability (HRV) metrics**	**Labs**	**ROC AUC**
1	Detrended fluctuation analysis (DFA) *α*_1_, multiscale entropy (MSE)	Lactate, INR, creatinine	0.84
2	DFA *α*_1_, MSE, fuzzy entropy	Lactate, creatinine	0.83
3	DFA *α*_1_, MSE, average *R*‐*R* interval (interbeat interval duration)	INR, creatinine	0.81
4	DFA *α*_1_, fuzzy entropy, goodness of fit of power law fit, respiratory rate	Lactate	0.82
5	DFA *α*_1_, MSE, average *R*‐*R* interval, slope of power law fit, asymmetry index	None	0.80

### 3.2. Qualitative Analysis

A total of six nurses (all from the ED) and 20 physicians (13 emergency medicine, 5 internal medicine, and 2 critical care) consented to interviews of mean duration 28 min (range: 20–39) for nurses and 22 min (range: 13–41) for physicians.

Table 5 provides an overview of themes and examples of subthemes identified in the physician interviews. *Drivers* for physicians to use the SA tool included the potential for the tool to facilitate *communication* between services, the capacity to improve efficiency of care (i.e., saving *time*), ability to guide *decision-making* and initiate a second look at a patient, provide help in quantifying risk, and ease of integration into *routine practice* through existing EMR systems. *Barriers* to physicians using the tool included a need to understand/agree on use of the tool among team members, process concerns on how reports will be received and interpreted, additional time required to generate report, need for additional monitoring equipment, need for education (e.g., score interpretation, validation, clearly define population, and timing of use), need for manual data entry, and gaining buy‐in. When asked if they thought the tool would provide some benefit to their practice, 11 (55%) physicians said that they saw a “definite” or “potential” benefit, 7 (35%) were unsure, and 2 (10%) did not currently see a benefit; one MD wanted a clear recommendation for action rather than risk of deterioration and the other MD did not know how to interpret the definition of “deterioration,” which was initially not overtly present on the report (it was subsequently added). Results include all responses and do not factor in the change in report over the duration of the study.

**Table 5:** Physician assessment: definitions and subthemes (B, barrier; D, driver) related to implementation of the SA tool identified through interviews with emergency medicine (13 ED), internal medicine (5 INT), and critical care (2 ICU) physicians (total *n* = 20).

**Theme: definition**	**Example sub-theme (B/D)**	**Frequency (%)**
**Communication:** any exchange of information between providers relevant to implementation	Facilitate communication between services (e.g., help facilitate discussion with consulting services) (D)	7 (35%)
	Need to understand/agree on use of tool (e.g., both ED and consultants need to “speak the same language” such as agreeing on a cutoff to be admitted into the ICU) (B)	4 (20%)
**Resources:** any tangible or intangible asset relevant to implementation	Time (e.g., busy emergency department and extra time to generate report may mean it does not get done; tool could improve efficiency) (B/D)	7 (35%)
	Monitoring equipment (e.g., other sites or settings may not have monitors and other resources to implement the tool) (B)	3 (15%)
**Education:** topics related to education needs and areas of misinterpretation around the tool or report	Education on score interpretation/how to use the report (e.g., make sure people understand the tool and how it is helpful) (B)	10 (50.0)
	Want information on validation (e.g., need evidence that the tool is valid and reliable) (B)	11 (55%)
	Define population for use (e.g., unclear how patient is screened in to use the tool) (B)	7 (35%)
**Tool/data access:** issues about tool and data access required in order to use the tool	Integration with EMR (e.g., easy/fast access to tool) (D)	16 (80%)
	Careful use of pop‐ups (e.g. popups useful to flag high‐risk patients but frequent pop‐ups get ignored) (B/D)	12 (60%)
	Manually entering data seen as a barrier to use (B)	5 (25%)
**Decision-making:** issues related to using the SA tool in clinical decision‐making	Guide decision for where/whether to admit (e.g., could help distinguish level of care needed) (D)	6 (30%)
	May initiate a second look at patient (e.g., having data/risk score can trigger MD to take a second look at patient they thought was doing well and identify “blindspots” in gestalt) (D)	8 (40%)
	Quantifies risk/objective measure (e.g. helpful to see numerical values/objective measures) (D)	4 (20%)
**Changes in routine:** issues related to adapting routine practice to incorporate SA tool	Easy to integrate (e.g. not much needs to change and all data already gathered as standard in EMR sepsis pathway) (D)	5 (25%)
	Process concerns (e.g. questions about who and how reports will be received and interpreted and roles for nurses and MDs need to be clearly explained) (B)	4 (20%)
**Automaticity:** issues related to long‐term adaptation of practice	Once set in routine, hard to change (e.g., familiarity, people like to use things they know and are used to) (B)	4 (20%)
	Needs to become automatic in use (e.g., Type 1 (fast) vs. Type 2 (informed) decisions, needs to be part of Type 1 decisions) (D)	2 (10%)
**Timing**	Issues related to when physicians should receive/read report (e.g., trade‐off of having lab results and early decisions) (B)	6 (30%)
**Use for non-ED patients:** application of the SA report outside of the emergency department	Non‐ED ward use (e.g., targeted to ED but could be useful on ward and long stay patients) (D)	2 (10%)
	Long‐term trends in report ratings (e.g., using report to observe change in patient risk ratings for admitted patients) (D)	2 (10%)
**Other:** other issues that may affect implementation	Buy‐in from relevant stakeholders (e.g., ED physicians, nurses, and consulting services need to agree tool should be used) (B/D)	6 (30%)
	Institutional support/encouragement (e.g., having support from administration and influential colleagues) (D)	1 (5%)

Table 6 provides an overview of themes and examples of subthemes identified in the interviews with nurses. Nurses identified many similar themes around using the SA tool in practice as physicians. Drivers for nurses included the potential for the tool to facilitate *communication* between services, the minimal training/*education* needed for data entry, potential ease of *access* through current EMR, the potential to improve care by informing *decision-making*, may help prevent patients from being missed, having a user‐friendly interface and report design, and fitting in well with other processes already in place. However, barriers to nurses′ use of the tool included the need to manually enter data taking *time*, that data is not yet auto‐populated through EMR, need for more education regarding interpretation, use, and how it fits with current workflow, difficulty in changing routines, and need for buy‐in. When asked if they would be able to pass along the report to physicians, five (83%) said that they were comfortable in physically handing off the report, and two of these nurses were also comfortable interpreting the report for physicians. All nurses indicated that completing the report took approximately 5–10 min, or less.

**Table 6:** Nursing assessment: definitions and subthemes (B, barrier; D, driver) related to implementation of the SA tool identified through interviews with registered nurses (*n* = 6).

**Theme: definition**	**Example subtheme**	**Frequency (%)**
**Communication:** exchange of information between providers	Facilitate communication between services (e.g., help facilitate discussion with internal medicine or other consulting services) (D)	2 (33%)
**Resources:** any tangible or intangible asset relevant to implementation	Time (e.g., data entry is time consuming, time taken away from other tasks, and time is limited in the ED) (B)	6 (100%)
	Help from others to enter data (e.g. RN dictated data for RA to enter, personal care assistant or med students) (D)	5 (83%)
**Education:** topics related to education needs and misinterpretation/understanding around the tool/report	Education on score interpretation, how to use, and when to generate report (e.g., unsure if risk prediction is with or without intervention, unsure of best timing to generate report so that it is most useful) (B)	5 (83%)
	No/minimal training needed for data entry (e.g., interface is clear and straightforward) (D)	5 (83%)
**Access:** issues about tool/data access required	Auto‐populate information (e.g., manually entering data seen as a barrier to use) (B)	5 (83%)
	Tool access (e.g., easy access through desktop or EMR) (D)	2 (33%)
**Decision-making:** issues related to using the SA tool in clinical decision making	Tool could improve patient care (e.g., patient flow, prioritizing patient needs, and MD decision‐making) (D)	5 (83%)
	May prevent patients being missed (e.g., may miss things in busy ED, tool could help ensure MD gets all sepsis related information) (D)	2 (33%)
**Report design/contents:** design features of the SA application/report related to use	User‐friendly interface (e.g., tool itself is easy to use and data entry is straightforward) (D)	4 (67%)
	Like report design/information provided (e.g., synthesises patient data for sepsis and top of report highlights important information) (D)	4 (67%)
**Changes in routine:** issues related to adapting routine practice to incorporate SA tool	Importance of familiarity (e.g., already familiar with electronic data, need familiarity with SA data entry tool, and daily reminders) (B)	5 (83%)
	Fits with sepsis processes already in place (e.g. things being input already on nurse′s mind and already get electronic prompts for sepsis in EMR) (D)	3 (50%)
**Timing:** issues related to when nurses should generate the SA report and give to physicians (e.g. trade‐off between labs available and early care decisions) (B/D)		2 (33%)
**Use for non-ED patients:** application of the SA report outside of the emergency department	Non‐ED ward use (e.g., empower ward nurses by showing MD the report to advocate for sepsis workup) (D)	1 (17%)
	Use at other sites/other patient populations (e.g., tool maybe useful at other sites with patients at higher risk of sepsis) (D)	1 (17%)
**Nurse′s role:** issues related to the nurse′s perceptions of role in relation to implementation of the SA tool	Perceived value to patient care (e.g., nurses more willing to complete SA reports if they see it has a positive impact for patients) (B/D)	4 (67%)
	Willingness to complete reports (e.g., not something they are eager to take on but willing to do if asked/required) (B)	3 (50%)
**Deciding to complete report:** issues related to emotion/memory/other cognitive processes around completing reports	Choosing not to complete report (e.g., nurse may not complete report due to patient acuity/workload) (B)	3 (50%)
	Directives empower RN to complete reports independently (e.g. can order bloodwork and other required investigations without consulting MDs) (D)	1 (17%)
**Receptivity:** issues related to receptivity of adapting practice to include SA reports	Receptivity depends on ease of use (e.g., needs to be fast/simple for people to use it) (D)	3 (50%)
	Receptive if improve patient care (e.g., if proven useful to clinical decisions RNs will feel tool is worthwhile) (D)	3 (50%)

Throughout the study, the physician and nursing interview feedback relating to usability and usefulness were compiled and summarized (by NH with supervision from JB) and presented to the study steering committee (all authors), who made recommendations for changes to the SA user interface and report. The SA tool underwent four iterative evolutions. The number of changes recommended after each iteration decreased as the study progressed, beginning with 12, followed by four, four, and two with each version.

## 4. Discussion

We conducted a prospective, observational, two center, mixed‐methods implementation study to evaluate the feasibility, initial perceptions, and used experience of this deployment of a HRV‐based predictive clinical decision support tool, SA, which combines HRV and available labs to help risk‐stratify ED patients with suspected infection and inflammatory response by predicting their likelihood of short‐term (i.e., within 1–72 h) deterioration. As investigators continue to derive and validate predictive models, it is vital that we study the practicality, feasibility, and optimal means of implementing predictive clinical decision support tools at the bedside. In this mixed‐methods feasibility implementation study, we found that the SA tool could be feasibly deployed in real time, utilizing heart rate EKG data for use in HRV‐derived predictive model generation in over 90% patients. The qualitative assessment of both physicians and nurses found that most providers were comfortable with the use of the SA tool and the data provided from the tool was useful and interpretable. Based on the UCD feedback, the SA tool was refined through four iterative versions to improve the usability and usefulness of the report.

Disposition decision‐making in patients with sepsis is complex and high‐stakes. For patients obviously presenting with hypotension and shock, disposition decision‐making is straightforward, requiring ICU admission [12]. However, for patients presenting without overt evidence of shock or respiratory distress, optimizing disposition is critical, as inappropriate disposition is associated with worse patient outcomes and increased costs of care [6]. Existing risk stratification tools for patients with infection have poor prognostic accuracy [10]. HRV represents an underutilized method for risk stratification of future deterioration [13]. HRV with clinical and laboratory factors is superior to clinical or laboratory features alone [14]. HRV is utilized in various patient populations [24]. However, the application of HRV prospectively in busy environments such as the ED is challenging [25]. This study supports the feasibility of real‐time application of an HRV‐based prognostic clinical decision support tool in the ED.

The qualitative component of our study identified drivers and barriers to SA implementation and informed improvements to the SA report. We interviewed physicians and nurses, and both groups of providers expressed sentiments that endorsed the use of the tool. ED nurses felt the SA data entry interface was easy to use and believed they could obtain the reports to pass on to physicians. The ED physicians felt that the information in the SA report would be useful in their decision‐making around management and disposition of patients with suspected infection. Further, the UCD process improved the usability and perceived usefulness of the reports as evidenced by the decrease in required changes after each iteration. Both physicians and nurses identified important challenges around the use of the SA tool, including necessary resources that might be required to use the tool routinely and education around the tool and its use. These themes identified in the qualitative analysis may be broadly relevant for implementation of future predictive clinical decision support software tools.

Only 6% (four of the 71 patients) enrolled in our study ultimately met the primary outcome of deterioration within 72 h. This emphasizes how difficult real‐time decisions surrounding disposition of this population can be for ED physicians, often while performing multiple other tasks simultaneously. A larger study is required to further validate and improve the predictive models. Future interventional studies evaluating the experience of nurses and clinicians are required, with a clinical trial ultimately required to evaluate clinical and financial impact.

Interpretations of this study must include awareness of important limitations to the study. First, the study included a small sample size and limited number of outcomes from two centers, which precluded evaluation of prognostic accuracy. Second, while all patients were suspected/treated with infection, 30% did not have a confirmed source of infection. This reflects real‐world limitations in the management of suspected infection and sepsis in the ED, where the etiology of infection may be unclear [4]. The results did not factor in the evolving nature of the tool in the study. Finally, this study was impacted by the Coronavirus disease 2019 pandemic, which created enormous strain and unique working conditions for ED providers [26]. This significantly impacted capacity for enrollment and made implementation more difficult. Finally, this was a study of two EDs in the same city, and therefore, our results may differ when expanded to other jurisdictions.

## 5. Conclusion

In this prospective, two‐center, pilot observational Phase I feasibility implementation study of the SA tool, we found that application of the SA tool and subsequent capture of HRV data was feasible in ED patients with suspected infection. Qualitative data revealed that ED providers were comfortable using the SA tool and felt that the data provided were generally useful in decision‐making. Future larger studies of the SA tool are warranted, including interventional implementation studies with process evaluation, including how clinicians variably respond to predictive decision support.

## Ethics Statement

This study was approved by the Research Ethics Board at The Ottawa Hospital (Approval Number 20200804‐01H) and conforms to the provisions of the Declaration of Helsinki. As this study was a Pilot Phase I observational study with no impact on care, informed consent was not required.

## Conflicts of Interest

AJE Seely is the founder and CEO of Therapeutic Monitoring Systems, a company founded to commercialize variability‐derived clinical decision support tools developed in Dr. Seely′s Ottawa Hospital Research Institute laboratory. The rest of the authors declare no conflicts of interest.

## Author Contributions

Andrew J.E. Seely and Douglas P. Barnaby contributed equally as co‐first authors. Jamie C. Brehaut and Jeffrey J. Perry contributed equally as co‐senior authors.

## Funding

This work was supported by the Ottawa Hospital Academic Medical Organization.

## Data Availability

The data that support the findings of this study are available on request from the corresponding author. The data are not publicly available due to privacy or ethical restrictions.
